# ASPP2 Is Phosphorylated by CDK1 during Mitosis and Required for Pancreatic Cancer Cell Proliferation

**DOI:** 10.3390/cancers15225424

**Published:** 2023-11-15

**Authors:** Yi Xiao, Yuanhong Chen, Jianan Chen, Jixin Dong

**Affiliations:** Eppley Institute for Research in Cancer and Allied Diseases, Fred & Pamela Buffett Cancer Center, University of Nebraska Medical Center, Omaha, NE 68198, USA; yi.xiao@unmc.edu (Y.X.); cheny@unmc.edu (Y.C.); jchen@unmc.edu (J.C.)

**Keywords:** pancreatic cancer, ASPP2, CDK1, YAP, phosphorylation, p53, mitosis

## Abstract

**Simple Summary:**

Pancreatic cancer is one of the leading causes of cancer death. Identifying and elucidating important molecular mechanisms underlying pancreatic cancer cell proliferation will provide opportunities for therapeutic interventions. This study aims to examine the role of apoptosis-stimulating protein of p53-2 (ASPP2) in pancreatic cancer. We found that ASPP2 is phosphorylated by cyclin-dependent kinase 1 (CDK1) during mitosis. Results further indicate that depletion of ASPP2 attenuates pancreatic cancer cell growth in vitro and in vivo. Finally, our results show that ASPP2 markedly affects the transcriptome landscape of yes-associated protein (YAP)-related genes. These findings, for the first time, reveal the CDK1-mediated phosphorylation sites of ASPP2 during mitosis. These findings also demonstrate the essential role of ASPP2 in pancreatic cancer cell growth and identify the key targets for ASPP2-loss-induced growth remission.

**Abstract:**

(1) Background: pancreatic cancer is highly lethal. The role of apoptosis-stimulating protein of p53-2 (ASPP2) in this lethal disease remains unclear. This protein belongs to the ASPP family of p53 interacting proteins. Previous studies in this lab used phosphate-binding tag (Phos-tag) sodium dodecyl sulfate (SDS) polyacrylamide gels and identified a motility upshift of the ASPP family of proteins during mitosis. (2) Purpose: this study expands on previous findings to identify the detailed phosphorylation regulation of ASPP2 during mitosis, as well as the function of ASPP2 in pancreatic cancer. (3) Methods: the Phos-tag technique was used to investigate the phosphorylation mechanism of ASPP2 during mitosis. Phospho-specific antibodies were generated to validate the phosphorylation of ASPP2, and ASPP2-inducible expression cell lines were established to determine the role of ASPP2 in pancreatic cancer. RNA sequencing (RNA-Seq) was used to uncover the downstream targets of ASPP2. (4) Results: results demonstrate that ASPP2 is phosphorylated during mitosis by cyclin-dependent kinase 1 (CDK1) at sites S562 and S704. In vitro and in vivo results show that ASPP2 is required for pancreatic cancer growth. Furthermore, the expressions of yes-associated protein (YAP)-related genes are found to be dramatically altered by ASPP2 depletion. Together, these findings reveal the phosphorylation mechanism of ASPP2 during mitosis. Collectively, results strongly indicate that ASPP2 is a potential target for abating tumor cell growth in pancreatic cancer.

## 1. Introduction

The latest cancer statistics in the United States show that pancreatic cancer is the fourth-leading cause of cancer death in men and women, with a five-year survival rate of only 12% [1]. The dismal outcome is mainly due to an inability to detect the disease at an early stage, compounded by the ineffectiveness in controlling the progression of this disease [2]. Currently, surgery and chemotherapy are the mainstay in pancreatic cancer treatment [2]. Surgery, or resection, despite offering the best chance to overcome the disease, is only suitable for 15–20% of patients at diagnosis [1,2]. Chemotherapy, on the other hand, is commonly used on most patients, regardless of surgical eligibility [2,3]. The first-line systemic chemotherapy includes two combination regimens: 5-fluorouracil, leucovorin, irinotecan, and oxaliplatin (FOLFIRINOX), and gemcitabine plus nab-paclitaxel [2,3]. Although efforts have been made to improve the standard of care for pancreatic cancer patients, achieving marked gains in survival remains a challenge [1,4]. Over the years, targeted therapy has advanced precision medicine in cancer treatment and provided considerable survival benefits for some types of cancer [5]. It is therefore imperative to identify and understand the molecular mechanisms of pancreatic cancer to inform rational therapeutic targets, thus improving patient outcomes from this recalcitrant disease.

Apoptosis-stimulating protein of p53-2 (ASPP2), apoptosis-stimulating protein of p53-1 (ASPP1), and the inhibitory member of the ASPP family (iASPP) constitute the apoptosis-stimulating proteins of P53 (ASPP) family of proteins, which is also referred to as ankyrin repeat-, SH3 domain-, and proline-rich region-containing proteins (ASPPs) [6,7,8,9]. All three ASPP proteins (i.e., ASPP2, ASPP1, and iASPP) share a highly conserved C terminus that contains ankyrin repeats and SRC homology 3 (SH3) domains, through which they interact with the regulatory protein p53, also known as tumor protein P53 [9]. While ASPP1 and ASPP2 enhance the transactivation functions of the p53 family members (i.e., p53, p63, and p73), iASPP represses p53-mediated gene expression [8,10,11]. Of note, ASPP1 or ASPP2 overexpression induces the binding of p53 to the promoters of proapoptotic genes (i.e., *BAX* and *PIG3*), but not the promoters of cell-cycle arrest genes (i.e., *CDKN1A* and *MDM2*) [11,12]. In contrast, in the presence of ASPP1 and ASPP2, overexpression of iASPP dramatically reduces the p53-dependent transcription of *BAX*, but not *CDKN1A* [8]. As such, ASPP proteins could be considered master regulators of cell apoptosis and affect cellular response to DNA-damaging agents [13].

The ASPP family of proteins also serves as the regulatory subunits of protein phosphatase-1 catalytic subunits (PP1-c: PP1A, PP1B, PP1C). ASPP proteins are crucial for neuronal development in mammalian cells. They are also critical for eye patterning and wing development in *Drosophila* [14,15,16]. Moreover, ASPP proteins control important mitotic events [17,18,19,20]. For example, ASPP1/2-PP1A complexes dephosphorylate the kinetochore component Hec1 to control kinetochore–microtubule attachment during early mitosis. ASPP1/2-PP1A complexes also dephosphorylate the centrosome linker component C-Nap1 to promote centrosome linker reassembly at the end of mitosis [17,18]. Moreover, iASPP-PP1C dephosphorylates the midbody protein CEP55 to trigger cytokinesis [19]. In addition, by binding to the microtubule end-binding protein EB1 or the actin-based molecular motor Myo1c, iASPP contributes to cell cortex rigidity. Additional contributions of iASPP to mitosis include cell rounding and spindle positioning [20]. Previous work by Xiao et al. showed robust mitotic upshift in all ASPP proteins on Phos-tag SDS polyacrylamide gels [21]. Notwithstanding the involvement of ASPP proteins in many biological processes, the functions of ASPP proteins in pancreatic cancer are an emerging area of research and remain underexamined. Despite the strong implications of all three ASPPs playing roles in mitosis, how the ASPPs themselves are regulated during mitosis has been largely unknown.

ASPP2 is defined as a haploinsufficient tumor suppressor due to the fact that ASPP2 haplodeficiency in mice develops spontaneous tumors [22,23]. The ASPP2 heterozygous knockout mice also exhibit an increased rate of tumor formation upon radiation or carcinogen insults [23,24]. Studies have shown that downregulation of ASPP2 promotes cancer cell proliferation, motility, and chemo-resistance in some types of carcinomas [25,26,27,28]. Apart from directly activating p53, ASPP2 binds Ras and further enhances the apoptosis-stimulating function of p53 in cancer cells [29].

There are multiple mechanisms regarding ASPP2-mediated tumor repression that involve inhibiting aerobic glycolysis/the Warburg effect through suppression of WNT/β-catenin signaling [25]. ASPP2 may repress tumors by strengthening epithelial polarity at apical cell–cell junctions via interacting with PAR3 [30]. As another example, ASPP2 may repress tumor formation by restricting epithelial–mesenchymal transition through preventing Smad7 degradation or β-catenin-mediated *ZEB1* expression [31,32]. ASPP2 may also induce apoptosis via physically binding to Bcl-2 family members at the functional sites to release proapoptotic proteins [33], as well as multiple other mechanisms regarding ASPP2-mediated tumor repression. Moreover, ASPP2 physically interacts with the yes-associated protein (YAP) and transcriptional co-activator with PDZ binding motif (TAZ) [34,35,36,37]. ASPP2 and PP1 cooperate to dephosphorylate YAP/TAZ at inhibitory phosphorylation sites and promote nuclear localization of YAP/TAZ for target gene expression [36,37]. Importantly, YAP and TAZ are the effectors of the Hippo signaling pathway and give rise to many malignant attributes, including tumorigenesis, drug resistance, metastasis, immunosuppression, etc. [38,39].

Although the interconnectedness of ASPP2 and p53 endows the tumor-suppressive properties of ASPP2, the interaction of ASPP2 and YAP/TAZ could trigger a different effect. This alludes us to re-examine the function of ASPP2 in cancer. Another interesting finding led by Godin-Heymann et al. indicated that ASPP2 was phosphorylated by mitogen-activated protein kinase (MAPK) at S698 and S827 [40]. Nevertheless, how ASPP2 is phosphorylated during mitosis remains unknown. Given the strong mitotic shift of ASPP2 detected in our previous study [21], the aim of the current study was to reveal the mitotic phosphorylation sites of ASPP2.

This study first identified CDK1-dependent phosphorylation sites of ASPP2 during mitosis. ASPP2 was then shown to be overexpressed in pancreatic cancer and associated with decreased overall survival. Next, ASPP2 proved to be essential for pancreatic cancer growth in vitro and in vivo. Further, mitotic phosphorylation of ASPP2 was found to be important for cell proliferation. Finally, through using RNA sequencing (RNA-Seq) to examine the downstream targets of ASPP2, several top hits were found to be the YAP targets. Together, this study, for the first time, deciphers how phosphorylation of ASPP2 is regulated during mitosis. This study also reveals the essential role of ASPP2 in pancreatic cancer cell growth and the underlying mechanisms.

## 2. Materials and Methods

### 2.1. Cell Culture and Transfection, and Transduction

HEK293T, HEK293GP, HeLa, Capan-2, BxPC-3, PANC-1, HPAF-II, and Hs766T cells were purchased from the American Type Culture Collection (ATCC). The human pancreatic nestin-expressing cells (HPNE, also known as hTERT-HPNE) expressing the catalytic subunit of human telomerase (hTERT) [41] were generated by Dr. Michel Ouellette (University of Nebraska Medical Center). S2.013, Colo-357, and T3M-4 were kind gifts from Dr. Michael (Tony) Hollingsworth (University of Nebraska Medical Center) and cultured as previously described [21,42]. All other cells were cultured according to the American Type Culture Collection (ATCC) instructions.

### 2.2. Anti-Mitotic Drugs and Transfection Agents

Nocodazole and taxol (Selleck Chemicals, Houston, TX, USA) were used at concentrations of 100 ng/mL and 100 nM, respectively, overnight or longer depending on the cell types to enrich the mitotic cells. The CDK1 inhibitor RO3306 (ENZO Life Sciences, Farmingdale, NY, USA), the CDK1/2/5 inhibitor purvalanol A (Selleck Chemicals), and the aurora inhibitor VX680 (Selleck Chemicals) were used as previously described [21]. MG132 (Santa Cruz Biotechnology, Dallas, TX, USA) was added along with the kinase inhibitors at a concentration of 25 μM to prevent mitotic exit.

Attractene (Qiagen, Hilden, Germany) was used as a transfection reagent. For retroviral packaging, VSV.G (Addgene, Watertown, MA, USA) was used. For lentiviral packaging, psPAX2 and pMD2.G (Addgene) were used. Transfections were conducted as per the manufacturer’s instructions. The viral transduction process was carried out as previously described [43].

### 2.3. Expression Constructs and RNA Interference

The human ASPP2 coding sequence (CDS) was obtained from Sino Biological Inc. and was further cloned into the pGEM-T vector (Promega, Madison, WI, USA) for mutagenesis. Point mutations (S562A and S704A, referred to as 2A) were generated using the Quik-Change Site-Directed PCR Mutagenesis Kit (Agilent, Santa Clara, CA, USA) and verified by Sanger sequencing.

The wild-type (WT) or mutated ASPP2 (2A) cDNA was cloned to the retroviral Tet-All (TetOn) vector [44] to establish cell lines with ASPP2-inducible overexpression. Cells transduced with Tet-All vectors were cultured in Tet-free medium with Tet system-approved fetal bovine serum (Clontech Laboratories, Mountain View, CA, USA). Vectors pcDNA3-CDK1-AF and GFP-Cyclin B1-R42A were from Addgene.

To generate ASPP2-inducible knockdown constructs, shRNA oligos were designed, annealed, digested, and inserted into the pLKO-TetOn vector (Addgene) as per the manufacturer’s instructions.

The ASPP2 shRNA targeting sequences were as follows:

- shASPP2-A: GTCTAGTAAATAGGATCATTT.
- shASPP2-B: GAAATCCAGAATCCATATTTA.

### 2.4. Phos-Tag, Western Blotting, and Peptide Blocking

The Phos-tag gel was made with 10 μM Phos-tag (Wako Pure Chemical Industries, Richmond, VA, USA) and 100 μM MnCl_2_ in SDS-acrylamide gel, and used as described in [45]. Western blotting and lambda phosphatase treatment assays were completed as described in [43]. Peptide blocking was performed following the Abcam website protocol (https://www.abcam.com/protocols/blocking-with-immunizing-peptide-protocol-peptide-competition, accessed on 6 December 2021).

### 2.5. Recombinant Protein Purification and In Vitro Kinase Assay

Part of the ASPP2-WT or ASPP2-2A cDNA (from 1768bp to 2871bp) was cloned to the previously mentioned pGEX-5X1-MCS construct expressing GST-tagged proteins [21]. The recombinant protein purification was completed as described [46]. Purified GST-ASPP2-WT and GST-ASPP2-2A were incubated with or without 150 ng cyclin B1-CDK1 kinase complex (SignalChem, Richmond, BC, Canada) at 30 °C for 45 min to allow for substrate phosphorylation. Later, an SDS loading buffer was added, and samples were denatured at 95 °C for 10 min followed by western blot analysis.

### 2.6. Antibodies

Rabbit polyclonal phospho-specific antibodies against ASPP2 S562 and S704 were generated and purified by AbMart, Inc. (Shanghai, China). The peptides used for immunizing rabbits were PRVLLSPSIPS (S562) and IPRPLSPTKLL (S704). The corresponding non-phosphorylated peptides were also synthesized and used for peptide blocking.

Anti-cyclin B1, β-actin, ASPP1, ASPP2, and iASPP antibodies were purchased from Santa Cruz Biotechnology. The anti-GST antibody was purchased from Bethyl Laboratories. The anti-Ki-67 antibody was from Pierce. Anti-CDK1, anti-YAP S127, anti-YAP, and anti-YAP conjugated antibodies were from Cell Signaling Technology (Danvers, MA, USA). Antibodies were used at 1:100–1:5000 dilutions as needed.

### 2.7. Cell Proliferation

On day one, 25,000 Capan-2, 50,000 BxPC-3, 20,000 PANC-1, and 5000 S2.013 cells were seeded in doxycycline-added medium (2 μg/mL). At indicated time points, cells were trypsinized and mixed with trypan blue (HyClone, Seattle, WA, USA) in a one-to-one ratio. The cell number was counted using the Countess II (Invitrogen, Waltham, MA, USA).

### 2.8. Immunofluorescence Staining and Confocal Microscopy

S2.013 cells were fixed with 4% paraformaldehyde for 20 min at 37 °C. The staining of YAP was completed per the manufacturer’s protocol (Cell Signaling Technology). The slides were mounted with DAPI (Invitrogen). The images were captured using a Zeiss 710 confocal laser scanning microscope (Carl Zeiss, Jena, Germany).

### 2.9. RNA Isolation, Reverse Transcription, and RT-PCR

RNA was extracted [47] and reverse transcribed into cDNA for RT-PCR, as previously described [43]. The RT primers used were as follows.

ASPP2: forward: TGGTGTGGCTCTGAACGTC; reverse: CCACTCACAATGTCCCTGCC.

YAP: forward: GTTACCAACACTGGAGCAGGATG; reverse: TCGAGAGTGATAGGTGCCACTG.

AREG: forward: TGATACTCGGCTCAGGCCA; reverse: AATGGTTCACGCTTCCCAGA.

CCND1: forward: ATCAAGTGTGACCCGGACTG; reverse: CTTGGGGTCCATGTTCTGCT.

CYR61: forward: CGAGGTGGAGTTGACGAGAA; reverse: TCCATTCCAAAAACAGGGAGC.

SOX9: forward: TCTGAACGAGAGCGAGAAGC; reverse: CCGTTCTTCACCGACTTCCT.

LIF: forward: CCACCCATGTCACAACAACC; reverse: CCCTGGGCTGTGTAATAGAGAA.

PGAM1: forward: TCTGCTAATCCCAGTCGGTG; reverse: CATAGCCAGCATCTCGTAGC.

SEMA7A: forward: CCACCTAAGGAGCGGACCC; reverse: AGTTCTCGCAGTCCCGCTTA.

EMP1: forward: ATATGCCAGTGAAGATGCCCT; reverse: TGTAGATGGACACCCCCACA.

TFAM: forward: GCGTTTCTCCGAAGCATGTG; reverse: CTGTAGTTTTTGCATCTGGGTTC.

SERPINE2: forward: CGATTTAACCCGAGCGAGCA; reverse: TTCCACCAAGGACGACCTGC.

FBN1: forward: CCCGGATTTACCCAACACCA; reverse: TCACACTCGTCCACGTCTTC.

MXD4: forward: GAACTCCCTGCTGATCCTGCT; reverse: GAGTTTGGCTCGTCTGTGCTT.

SELL: forward: GACCAAGCAAAGCCATGATATT; reverse: AACAGAGCATTGTCCACCCC.

SPOCK1: forward: CGAGGATGCGAACAGAGTCA; reverse: GATGCTAGTGTCAAACCTGCC.

### 2.10. RNA-Seq

In three independent dishes, S2.013 (i-shControl or i-shASPP2-A) cells were seeded in doxycycline (2 μg/mL)-containing medium. Five days later, all six dishes of cells were harvested for RNA extraction. At least 1 μg of RNA was obtained from each sample for standard RNA sequencing (RNA-Seq).

RNA-Seq library preparation, quality control, and Illumina next-generation sequencing were performed with Genewiz (Azenta, Burlington, MA, USA). The raw data were trimmed using Trimmomatic v.0.36. The trimmed reads were mapped to the homo sapiens GRCh38 with the External RNA Controls Consortium (ERCC) genes reference genome on ENSEMBL using the STAR aligner v.2.5.2b.

Gene hit counts were calculated using featureCounts from the Subread package v.1.5.2.

Differential gene analysis was completed using DESeq2. The Wald test was used to generate *p* values and log2 fold changes. Genes with an adjusted *p* value < 0.05 and absolute log2 fold change > 1 were considered to be differentially expressed genes.

The heatmap and volcano plot were generated as described in [48]. Gene ontology (GO) analysis was completed using the Gene Ontology Annotation (GOA) human GO list to cluster the sets of genes. The enrichment of GO terms was implemented using the GeneSCF v.1.1-p2 software; the Fisher’s exact test was used.

### 2.11. Xenograft Mouse Model

Eight-week-old male athymic nude mice (Jackson Laboratory) were used for xenograft assays. The S2.013 cells (i-shControl and i-shASPP2, 2.5 × 10^6^) were suspended with PBS and injected subcutaneously into either flank of the mouse. Seven animals were used per group. Six days after inoculation, doxycycline (1 mg/mL in 5% sucrose water) was added to their diet. Tumor size was measured using a caliper twice a week, and tumor volume (V) was calculated with the formula V = 0.5 × length × width^2^. Mice were euthanized by CO_2_ inhalation at the end of the experiments, and the tumors were resected for imaging. All animals were housed in pathogen-free facilities. All animal experiments were approved by the University of Nebraska Medical Center Institutional Animal Care and Use Committee.

### 2.12. Statistical Analysis

Unless otherwise noted, a two-tailed Student’s *t* test was used to assess the statistical significance. A *p* value < 0.05 was considered statistically significant.

## 3. Results

### 3.1. The ASPP Family of Proteins Are Phosphorylated during Mitosis

**Figure 1:**
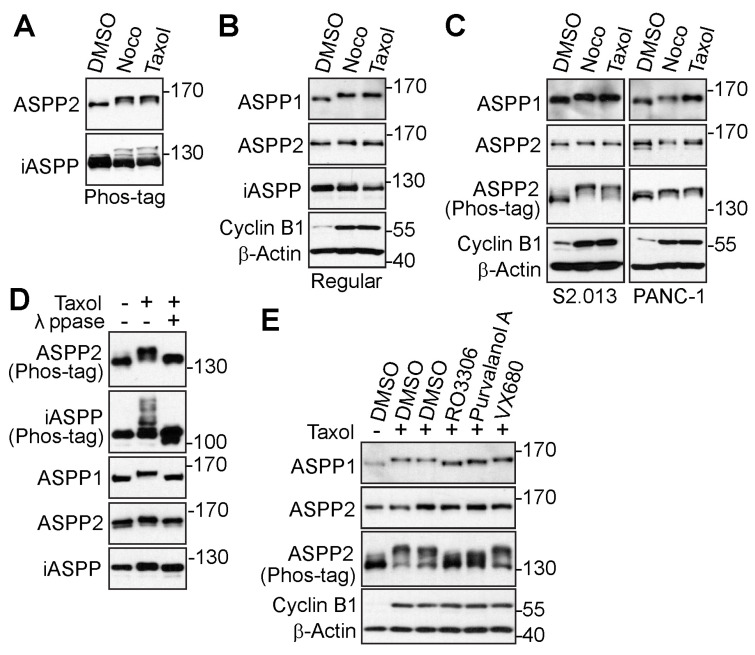
The ASPP family of proteins is phosphorylated during mitotic arrest. (**A**,**B**) HeLa cells were treated with dimethyl sulfoxide (DMSO), nocodazole (Noco, 100 ng/mL), or taxol (100 nM) overnight. Total cell lysates were electrophoresed on Phos-tag (**A**) or regular (**B**) SDS polyacrylamide gels and probed with indicated antibodies. The increased Cyclin B1 level indicates cells in G2/M phase. (**C**) S2.013 and PANC-1 cells were treated as in (**A**). Total cell lysates were probed with the indicated antibodies. (**D**) HeLa cells were treated with DMSO or taxol, and total cell lysates were further incubated with (+) or without (−) lambda phosphatase (λ ppase) prior to western blotting. (**E**) HeLa cells were treated with taxol overnight and subjected to the indicated kinase inhibitors for 1.5 h. MG132 was added along with the kinase inhibitors to prevent mitotic exit. The lysates were harvested for western blotting. The uncropped bolts are shown in Supplementary Materials.

Using a Phos-tag system, our previous study screened a series of phosphatases and regulatory subunits subjected to phosphorylation modification during mitosis [21]. All three ASPP proteins were discovered to be up-shifted on Phos-tag gels by anti-tubulin agents, nocodazole, and taxol, which induced mitotic arrest [21]. To further confirm that the mitotic shift was caused by phosphorylation, cells were treated with anti-microtubule drugs again. The proteins were examined on Phos-tag or regular SDS polyacrylamide gels. As clearly seen on Phos-tag gels, ASPP2 and iASPP were upshifted in nocodazole- or taxol-treated HeLa cells (Figure 1A). Whereas the shift of ASPP2 or iASPP was faint on the gel, the mitotic arrest-induced upshift of ASPP1 was easily observed (Figure 1B). The up-shifted bands of ASPP1 and ASPP2 were also seen in pancreatic cancer cell lines (Figure 1C).

To rule out the possibility that the motility upshift was caused by other post-translational modifications, the taxol-induced samples were incubated with or without λ-phosphatase. The upshifted bands of all three ASPPs were shown to return to their original states from λ-phosphatase treatment (Figure 1D), indicating that the reduced movement on the SDS polyacrylamide gels was due to phosphorylation modification. To identify the mitotic kinases for ASPP2 phosphorylation, the protein post-translational modification database (www.phosphosite.org, accessed on 11 September 2020) was used, and it was found that ASPP2 harbored potential CDK1-mediated (S/T-P) and aurora kinases-mediated (R-X-S/T).

phosphorylation sites [49,50]. Given this discovery, the arrested cells were treated with CDK1 inhibitors (RO3306 or purvalanol A) or an aurora kinase inhibitor (VX680). Results showed the CDK1 inhibitors dramatically blocked the mobility upshift of ASPP2, while the aurora kinase inhibitor failed to do so (Figure 1E). Given the significant homology between ASPP1 and ASPP2, the mobility shift of ASPP1 was also examined under similar conditions. Interestingly, results showed the up-shifted bands of ASPP1 could also be reversed by CDK1 inhibitors. Therefore, these findings suggest the ASPP family of proteins is phosphorylated during mitosis, most likely by CDK1.

### 3.2. ASPP2 Is Phosphorylated at S562 and S704 by CDK1 during Mitosis

**Figure 2:**
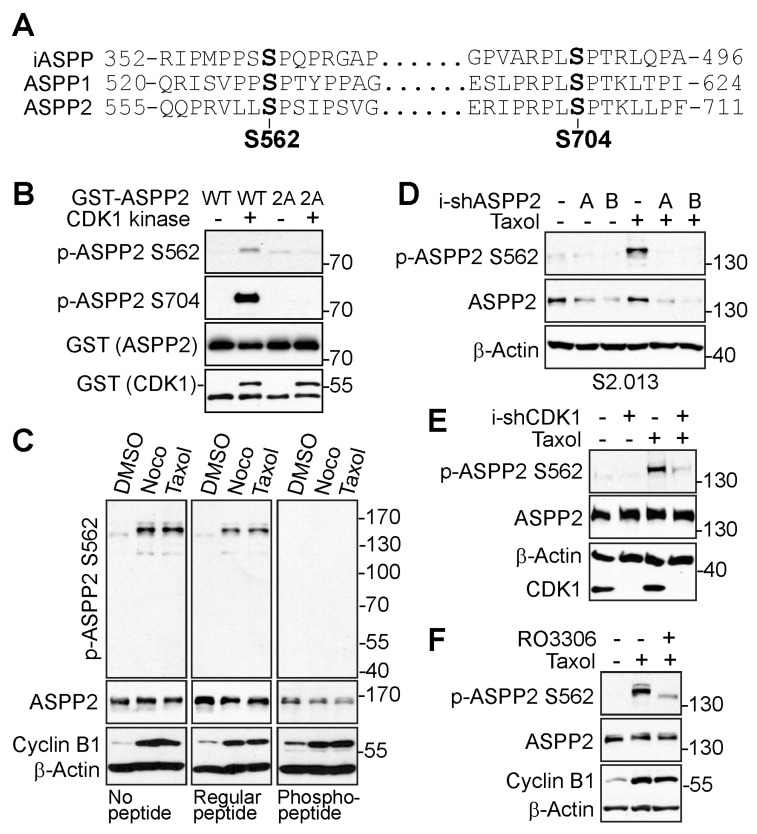
ASPP2 is phosphorylated at S562 and S704 by CDK1 during mitosis. (**A**) Conservation of S562 and S704 among the ASPP family proteins. (**B**) Partial ASPP proteins (GST-ASPP2-WT or GST-ASPP2-2A, ASPP2 amino acids from 501 to 868) were purified from bacteria for in vitro kinase assay. Purified proteins were incubated with or without purified CDK1/cyclin B1 complex prior to western blotting for detection of specific phospho-sites. 2A: S562A/S704A. (**C**) Hela cells were treated with nocodazole or taxol overnight. Lysates were electrophoresed on SDS polyacrylamide gels with non-phospho-peptide or specific phospho-peptide blocking and probed with S562 phospho-antibody. (**D**) ASPP2 inducible knockdown S2.013 cells along with the control cells were treated with taxol for 24 h prior to harvesting for detection of phosphorylation at S562. (**E**) CDK1 inducible knockdown HeLa cells along with the control cells were treated with taxol as indicated and lysates were harvested for western blot analysis. (**F**) HeLa cells were treated with Taxol alone or together with CDK1 inhibitor (RO3306) as indicated for western blot analysis. The uncropped bolts are shown in Supplementary Materials.

Based on the phosphorylation database, S562 and S704 are the potential ASPP2 phosphorylation sites possessing the consensus sequence for CDK1 phosphorylation. Because ASPP proteins are highly homologous, the conservation of these sites was checked in all three ASPP proteins using a sequence alignment tool, Clustal Omega (https://www.ebi.ac.uk/Tools/msa/clustalo/, accessed on 19 January 2023). As indicated in Figure 2A, these phosphorylation sites were conserved among the ASPP proteins.

Next, phospho-specific antibodies against ASPP2-S562 and ASPP2-S704 were generated to investigate the phosphorylation sites of ASPP2 during mitosis. An in vitro kinase assay was first performed to examine whether these two sites could be phosphorylated by CDK1 in vitro. As shown in Figure 2B, CDK1 directly phosphorylated the wild-type ASPP2 (GST-ASPP2-WT) at S562 and S704 in vitro, but not the phosphorylation-deficient ASPP2 (GST-ASPP2-2A: S562A/S704A).

Although the phospho-antibody against S704 detected a robust phosphorylation signal of ASPP2 in vitro, it was not ideal to examine the site-specific phosphorylation in the cells due to the lack of specificity. Hence, the following experiments focused on the phosphorylation of ASPP2 at S562 in cells. To verify the specificity of the S562 phospho-antibody, peptide blocking experiments first were performed. Incubation with the S562-specific phospho-peptide, but not the regular non-phospho-peptide, completely abolished the phospho-signals (Figure 2C). Next, the study aimed to determine whether the phospho-signal could be repressed by ASPP2 depletion. As expected, the mitotic arrest-induced phosphorylation of ASPP2 at S562 was lost by ASPP2 knockdown (Figure 2D). To investigate the CDK1-dependent phosphorylation of S562 in cells, the influence of CDK1 on S562 phosphorylation was tested by inhibiting CDK1. Both genetic depletion (Figure 2E) and pharmaceutical inactivation (Figure 2F) of CDK1 greatly suppressed the S562 phospho-signals during mitotic arrest. Together, these data support that CDK1 mediates the phosphorylation of ASPP2 during mitosis at sites S562 and S704.

### 3.3. ASPP2 Is Overexpressed and Required for Pancreatic Cancer Growth

**Figure 3:**
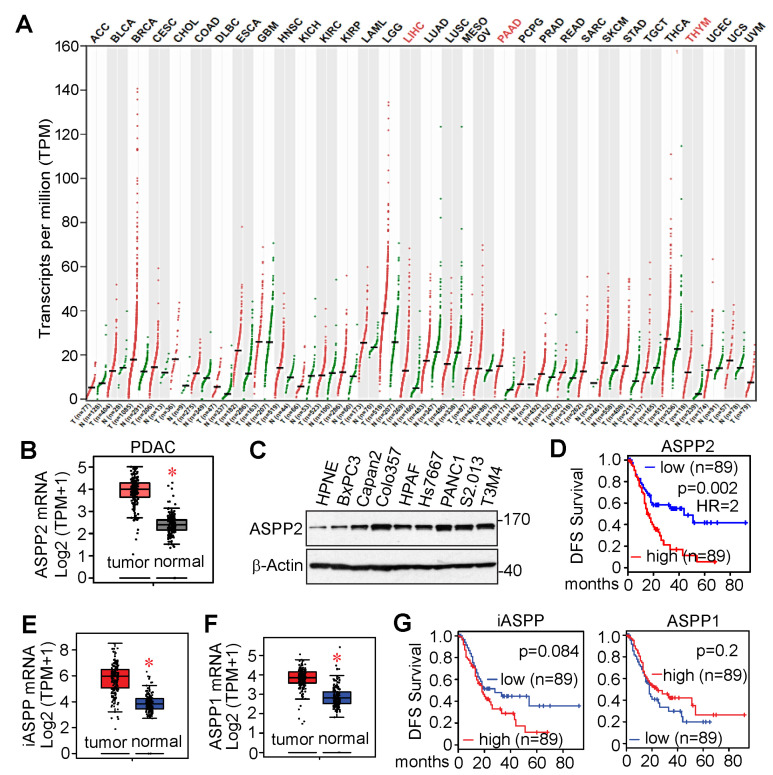
ASPP2 is overexpressed in pancreatic cancer and associated with decreased survival. (**A**) ASPP2 expression (transcript per million, TPM) across different types of cancers. ASPP2-overexpressed cancer types include liver hepatocellular carcinoma (LIHC), pancreatic adenocarcinoma (PAAD), and thymoma (THYM), and are highlighted in red. (**B**) mRNA levels of ASPP2 are increased in pancreatic cancers (n = 179) compared to normal tissue (n = 171). (**C**) ASPP2 is overexpressed in pancreatic cancer cells compared to the immortalized pancreatic HPNE cells by western blot analysis. (**D**) Elevated ASPP2 expression is associated with reduced disease-free survival (DFS) in pancreatic cancer. (**E**,**F**) mRNA levels of ASPP1 (**E**) and iASPP (**F**) are increased in pancreatic cancer. Asterisks indicate *p* < 0.01. (**G**) Increased ASPP1 or iASPP does not affect pancreatic cancer patient outcome. The expression and survival analyses are based on the TCGA or GTEx data using the online bioinformatic tool GEPIA2. The uncropped bolts are shown in Supplementary Materials. Asterisks indicate *p* < 0.01.

According to The Cancer Genome Atlas (TCGA) and Genotype Tissue Expression (GTEx) data from the public cancer portal Gene Expression Profiling Interactive Analysis 2 (GEPIA2) [51], ASPP2 mRNA expression is greater in liver hepatocellular carcinoma (LIHC), pancreatic adenocarcinoma (PAAD), and thymoma (THYM) (Figure 3A,B). ASPP2 is also highly expressed in pancreatic cancer cell lines compared to the immortalized pancreatic HPNE cells (Figure 3C) [41]. Furthermore, increased expression of ASPP2 resulted in poor disease-free survival (Figure 3D). ASPP1 and iASPP are also upregulated in pancreatic tumors (Figure 3E,F). However, unlike ASPP2, increased expression of ASPP1 or iASPP does not correlate with disease-free survival of pancreatic cancer patients (Figure 3G).

**Figure 4:**
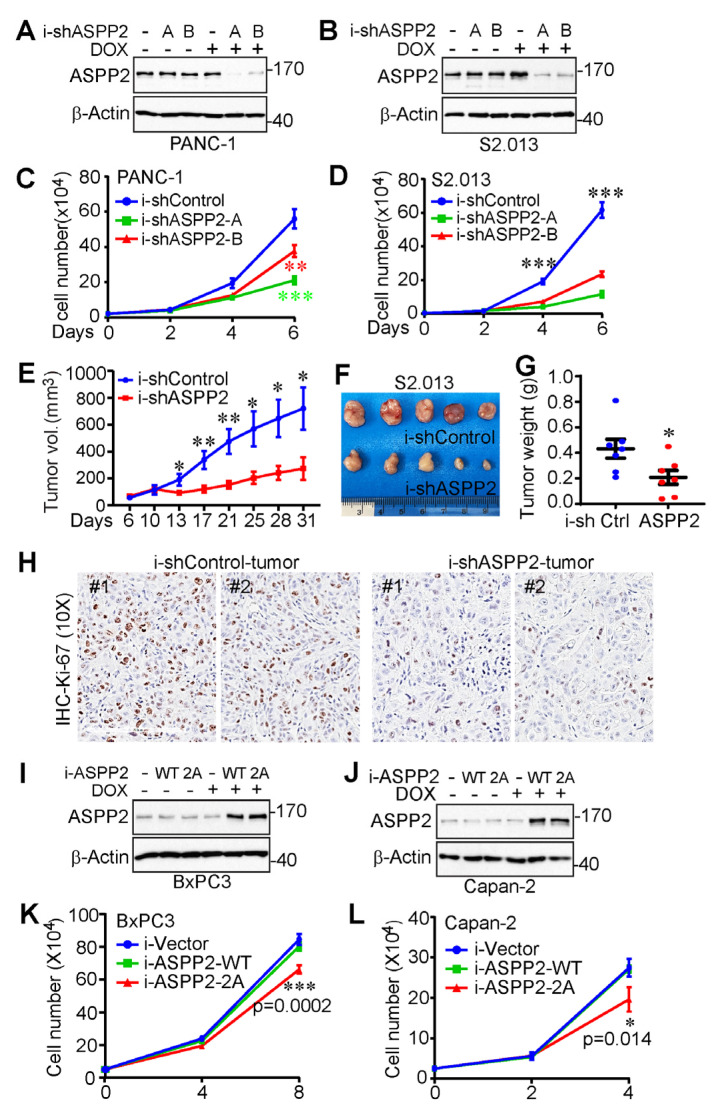
ASPP2 is required for pancreatic cancer cell proliferation. (**A**,**B**) Establishment of ASPP2-inducible knockdown PANC-1 (**A**) and S2.013 (**B**) cell lines with a TetOn inducible system. (**C**,**D**) Cell proliferation assays in PANC-1 (**C**) and S2.013 (**D**) cells from A and B. ***: *p* = 0.00054 (shRNA-A vs. control) and *p* = 0.0085 (shRNA-B vs. control) in panel C. ***: *p* = 6.99 × 10^−5^ (shRNA-A vs. control) and 0.00019 (shRNA-B vs. control) in panel D. Data are expressed as the mean ± SD of three independent experiments. (**E**–**H**) Tumor growth curve (**E**), representative images (**F**), end-point tumor weight (**G**), and Ki-67 staining (**H**) of ASPP2-depleted S2.013 cells as well as the control counterparts in xenograft mouse models. Cells were subcutaneously inoculated into athymic nude mice (seven mice in each group). Doxycycline (1 mg/mL) was added to the diet on day six. Data are expressed as the mean ± SEM. *: *p* < 0.05; **: *p* < 0.01. (**I**,**J**) Establishment of ASPP2-inducible overexpression Capan-2 (**I**) and BxPC-3 (**J**) cell lines with a TetOn inducible system. (**K**,**L**) Cell proliferation assays in Capan-2 (**K**) and BxPC-3 (**L**) cells from I and J. Statistical significance between the ASPP2-overexpressed (ASPP2-WT and ASPP2-2A, 2A: S562A/S704A) cells is indicated by *p* values in the panels. Data are expressed as the mean ± SD of three independent experiments. Student’s *t* test was used to analyze the statistical significance of this figure. The uncropped bolts are shown in Supplementary Materials.

To determine the role of ASPP2 in pancreatic cancer cell growth, ASPP2 in PANC-1 and S2.013 cells was effectively depleted using a TetOn-inducible system (Figure 4A,B). As observed in Figure 4C,D, pancreatic cancer cell proliferation was attenuated by a deficiency of ASPP2.

To examine the influence of ASPP2 on tumor growth in vivo, ASPP2-knockdown S2.013 cells were also inoculated into athymic nude mice, as well as their control counterparts. After allowing the tumor to grow for six days, doxycycline was added to their diet to initiate ASPP2 knockdown. As illustrated by the tumor growth curve, the end-point tumor weight, and the representative tumor images in Figure 4E–G, ASPP2 depletion clearly restricted tumor growth in the animal models. As expected, Ki-67 staining revealed that cell proliferation was significantly reduced in ASPP2 knockdown tumors compared to control tumors (Figure 4H).

In addition, ASPP2-inducible overexpression cell lines were established in order to investigate whether ASPP2 mitotic phosphorylation could affect cancer cell growth (Figure 4I,J). Inducible overexpression of wild-type ASPP2 was not found to impact the growth rate, whereas phosphorylation-deficient ASPP2 exerted a moderate dominant negative effect on pancreatic cancer cell proliferation (Figure 4K,L). These results indicate ASPP2 is upregulated in pancreatic cancer and required for pancreatic cancer growth in vitro and in vivo.

### 3.4. ASPP2 Regulates the Expression of YAP-Associated Genes

**Figure 5:**
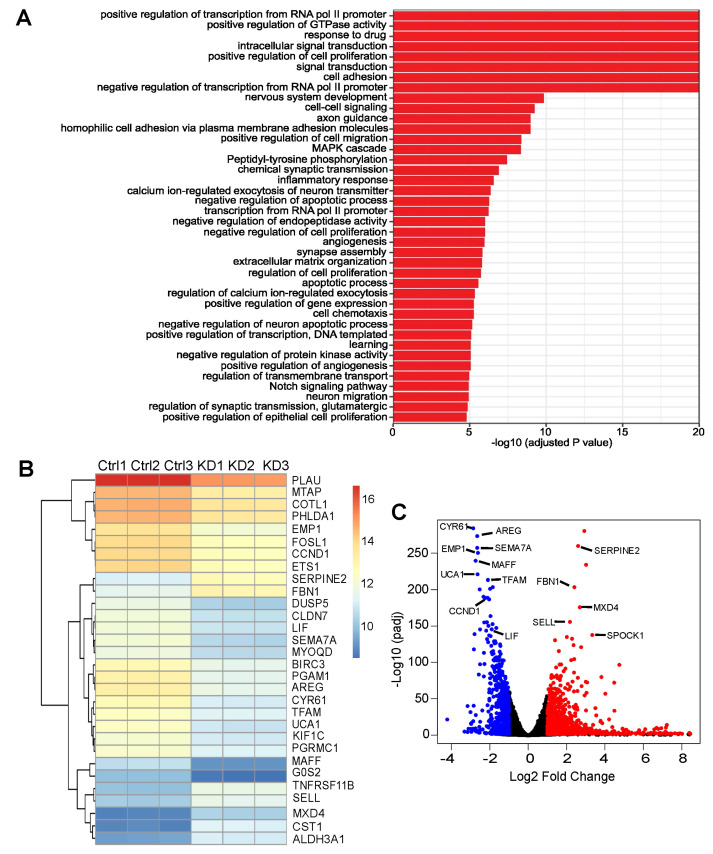
RNA sequencing (RNA-Seq) analysis identifies differentially expressed genes and biological processes altered by ASPP2 depletion. (**A**) Top 40 gene ontology (GO) terms altered by ASPP2 depletion in S2.013 cells. Significantly differentially expressed genes were clustered according to the gene ontology annotation (GOA) human GO list and the enrichment of GO terms was examined using Fisher’s exact test. (**B**) A heatmap showing the expression profile of the top 30 differentially expressed genes. Log2 transformed expression values are listed by the heatmap. (**C**) A volcano plot visualizing the global transcriptional change across the ASPP2 knockdown and control cells. Significantly downregulated genes are indicated by blue dots, and significantly upregulated genes are plotted by red dots.

To examine the downstream mechanism accounting for how ASPP2 deficiency is able to cause a reduction in tumor growth in pancreatic cancer, RNA-Seq was performed, and the differentially expressed genes in control and ASPP2-depleted S2.013 cells were compared. Based on the gene ontology (GO) analysis (Figure 5A), cell proliferation-related biological processes were listed among the top 40 significantly altered GO terms, including positive regulation of cell proliferation (GO:0008284), negative regulation of cell proliferation (GO:0008285), and regulation of cell proliferation (GO:0042127). Details of differentially expressed genes were further scrutinized to gain insights into the potential players downstream of ASPP2 affecting cell proliferation. Out of 18,133 genes sequenced, 1968 genes were identified as significantly changed genes, with 558 genes downregulated and 1410 genes upregulated by ASPP2 knockdown (Supplementary Materials).

**Figure 6:**
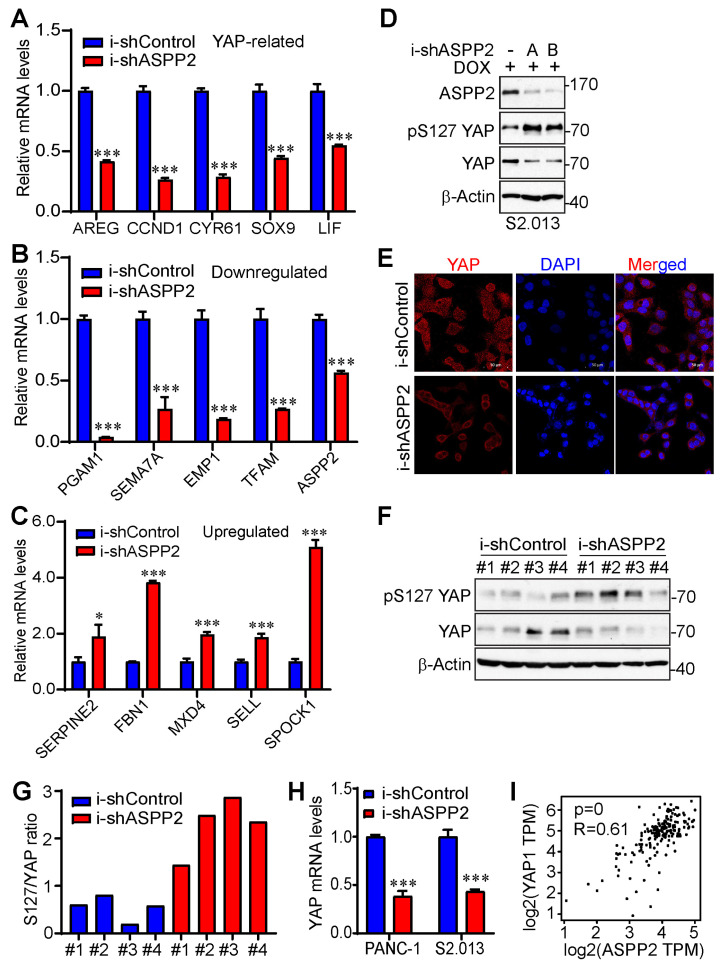
ASPP2 promotes YAP activity. (**A**–**C**) RT-PCR validating YAP-related genes (**A**), downregulated genes (**B**), and upregulated genes (**C**) by ASPP2 depletion from the top differentially expressed gene list. *: *p* < 0.05; ***: *p* < 0.001 (Student’s *t* test). Data are expressed as the mean ± SD of three independent experiments. (**D**) Western blot analysis showing increased phosphorylation of YAP at S127 and decreased YAP total proteins in ASPP2-depleted S2.013 cells. (**E**) Representative immunofluorescence staining images illustrating cytosolic retention of YAP in ASPP2 knockdown S2.013 cells. YAP was stained by an Alexa Fluor^®^ 647 anti-YAP antibody indicated in red. The nucleus was stained by DAPI and is marked by the blue color. Scale bar: 50 µm. (**F**,**G**) YAP activity was reduced in ASPP2 knockdown tumors. Tumor samples (#1–#4) were probed with the indicated antibodies (**F**). Quantification was performed using ImageJ (**G**). (**H**) RT-PCR showing the relative mRNA expression of YAP in ASPP2 knockdown and control cell lines. ***: *p* = 0.000048 in PANC-1 and *p* = 0.00017 in S2.013 (Student’s *t* test). Data are expressed as the mean ± SD from three independent experiments. (**I**) Correlation analysis of ASPP2 and YAP mRNA expression based on the TCGA database using GEPIA2. The uncropped bolts are shown in Supplementary Materials.

As shown by a heatmap listing the top 30 differentially expressed genes and a volcano plot illustrating global transcriptionally changed genes (Figure 5B,C), several YAP-related genes were markedly downregulated. These genes include *CCND1*, *LIF*, *AREG*, and *CYR61*. Except for LIF, which acts upstream of YAP and promotes YAP activity by restricting the phosphorylation of the upstream inhibitory kinases, including mammalian Ste20-like kinase 1/2 (MST1/2) and large tumor suppressor kinase 1 (LATS1) [52], *CCND1*, *AREG,* and *CYR61* are the known YAP targets [53,54,55]. Additionally, *SOX9*, another important target of YAP [56], was also downregulated and listed among the top 70 differentially expressed genes. Next, RT-PCR was performed to validate RNA-seq results. The YAP-related genes were first examined, revealing that the mRNA levels of all five aforementioned YAP-associated genes were reduced upon ASPP2 depletion (Figure 6A). Several downregulated and upregulated genes from the top list were also tested. Results confirmed that, indeed, the expressions of *PGAM1*, *SEMA7A*, *EMP1*, and *TFAM* were attenuated, whereas the expressions of *SERPINE2*, *FBN1*, *MXD4*, *SELL*, and *SPOCK1* were enhanced by the loss of ASPP2 (Figure 6B,C).

Considering the conspicuous change of YAP-targeted genes by ASPP2 silencing, the study went on to examine whether ASPP2 regulates YAP activity in pancreatic cancer cells. Results showed that the phosphorylation of YAP at S127 was elevated, and the YAP total levels were diminished by ASPP2 depletion (Figure 6D), which was consistent with a 2014 report by Royer et al. [36]. The knockdown of ASPP2 also triggered cytosolic retention of YAP (Figure 6E), an important mechanism for YAP inactivation and degradation. Reduced YAP activity (high p-S127 YAP/YAP ratio) was also detected in ASPP2 knockdown tumor samples (Figure 6F,G). Interestingly, a decrease in YAP mRNA expression was noted in ASPP2-depleted pancreatic cancer cell lines (Figure 6H). Further, there was a positive correlation between YAP and ASPP2 mRNA levels in pancreatic cancer samples from the TCGA database (Figure 6I). Overall, these data suggest that ASPP2 regulates the expression of multiple proliferation-related genes, and YAP is a key target of ASPP2 in pancreatic cancer.

### 3.5. RNA-Seq Identified Genes Correlating with ASPP2 Expression and Implying Negative Patient Outcome

**Figure 7:**
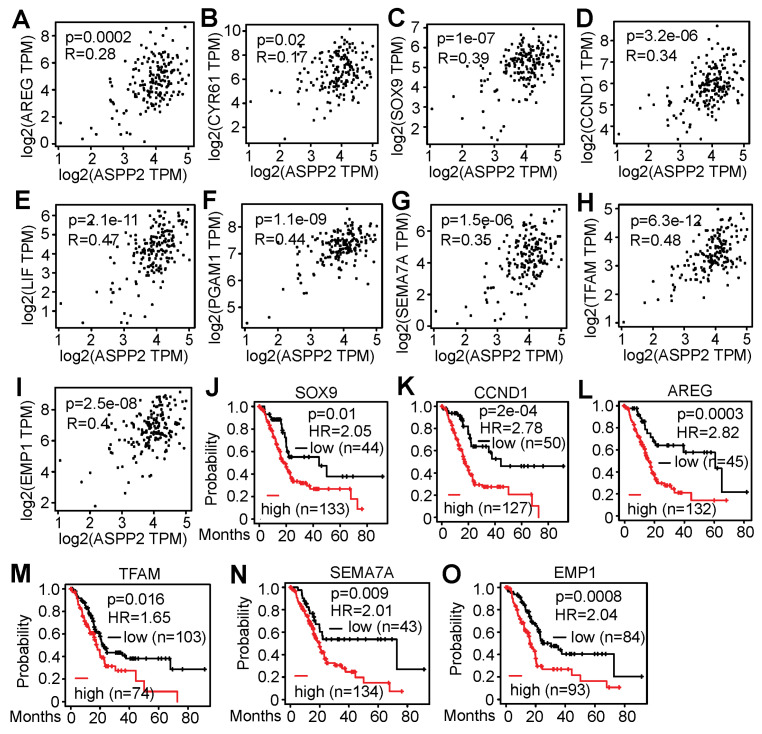
RNA-Seq identifies genes associated with ASPP2 expression and decreased patient survival. (A-I) Correlation analyses showing the positive association of ASPP2 with AREG (**A**), CYR61 (**B**), SOX9 (**C**), CCND1 (**D**), LIF (**E**), PGAM1 (**F**), SEMA7A (**G**), TFAM (**H**), and EMP1 (**I**) at mRNA levels. (**J**–**O**) Survival analyses indicating high mRNA levels of SOX9 (**J**), CCND1 (**K**), AREG (**L**), TFAM (**M**), SEMA7A (**N**), and EMP1 (**O**) imply reduced overall survival in pancreatic cancer patients. The data were generated using GEPIA2 using the TCGA databases.

This study further examined whether there is a correlation between ASPP2 and the validated genes in patient samples using the TCGA data from GEPIA2. The mRNA levels of many downregulated genes (i.e., *AREG*, *CYR61*, *SOX9*, *CCND1*, *LIF*, *PGAM1*, *SEMA7A*, *TFAM*, and *EMP1*) were found to be positively associated with ASPP2 expression (Figure 7A–I). Interestingly, elevated expression of most of these genes (i.e., *AREG*, *SOX9*, *CCND1*, *SEMA7A*, *TFAM*, and *EMP1*) correlated with unfavorable impacts on patient survival (Figure 7J–O). Therefore, RNA-Seq data from this study identify some candidate genes that positively correlate with ASPP2 at mRNA levels in pancreatic cancer patient samples and signify poor patient outcomes.

## 4. Discussion

Inspired by previous findings indicating the critical roles of the ASPP family of proteins in mitosis [17,18,19,20], our current study further unveiled how ASPP2 itself is regulated. Here, ASPP2 is demonstrated to be phosphorylated at S562 and S704 by CDK1 during mitosis. Intriguingly, S704 has been identified as a putative phosphorylation site of MAPK by Godin-Heymann et al. because it satisfied the criteria for the consensus sequence (also S/T-P) targeted by MAPK [40]. However, MAPK-dependent direct phosphorylation of S704 was not observed. In contrast, once we generated a phospho-specific antibody against this site, we detected a robust increase in S704 phosphorylation by CDK1 through an in vitro kinase assay. This result suggests that the phosphorylation of S704 is CDK1-dependent. Nevertheless, the possibility that S704 can also be phosphorylated by MAPK should not be excluded.

Another interesting finding was that phospho-deficient ASPP2 (S562A/S704A) only led to a moderate decrease in cell proliferation. Considering the homology among members of the ASPP family of proteins and the conservation of these two sites, there could be a functional redundancy among ASPP proteins to avoid the detrimental mitotic events caused by the hypophosphorylation of ASPP2 during mitosis. Further mutating the conserved sites in ASPP1/iASPP or depleting ASPP1/iASPP in ASPP2-mutated (ASPP2-2A) cells may assist in resolving this question.

Given their capabilities to enhance the transcriptional activity of p53, there is a mainstream belief that ASPP1 and ASPP2 are tumor suppressors. Be that as it may, we inspected ASPP1 and ASPP2 based on their abilities to activate YAP [36,57]. It was discovered that ASPP1 could bind to LATS1 and impede the interaction between YAP and LATS1, while the ASPP2-PP1 complex was found to directly dephosphorylate YAP at S127 to increase YAP activity. Additionally, data demonstrate that ASPP2 is associated with decreased patient survival and required for cell proliferation in pancreatic cancer, whereas ASPP1 failed to influence either. Although ASPP1 and ASPP2 share many similarities in that they activate p53 family proteins [11], YAP/TAZ [36,37,57], Ras [29], etc., they have disparate functions in pancreatic cancer and activate YAP through different mechanisms. Therefore, ASPP1 and ASPP2 are not “identical twins.” There is still much to uncover about their distinct roles. Apart from the fact that YAP, being an important downstream effector of ASPP2, could exert some effects on cancer cells that are completely opposite of tumor suppression, frequent mutation of *p53* in pancreatic cancer may also explain why lack of ASPP2 did not improve the tumor growth as expected. A *p53* mutation occurs in 50–70% of pancreatic cancers and may result in the loss of the tumor-suppressive function of p53 [58,59]. Our current study used *p53*-mutated PANC-1 and S2.013 cells in proliferation assays and found that ASPP2 deficiency impeded rather than boosted cell proliferation rate. In the future, it merits investigating whether the functions of the ASPP family of proteins are affected by the *p53* status in cancer.

Surprisingly, in our RNA-Seq data, we did not see a significant reduction in BAX or PIG3 mRNA by ASPP2 knockdown. *BAX* and *PIG3* are p53-targeted proapoptotic genes and can be induced by ASPP1 and ASPP2, according to past studies [11,12]. The difference in the findings could be due to the inactivation of p53 under normal conditions, which could lead to a low expression of BAX or PIG3 at the basal level, making it more difficult to detect a decrease in gene expression. This concern may be resolved by a simple experiment in which researchers can activate p53 using DNA damage agents in ASPP2-depleted cells and examine the mRNA levels of BAX and PIG3 again. Herein, we show that several YAP-targeted genes (i.e., *AREG*, *CCND1*, *CYR61*, and *SOX9*) are the top differentially expressed genes upon ASPP2 depletion. Consistent with previous findings [36], we observed increased phosphorylation of YAP at S127, reduced YAP total protein, and cytosolic retention of YAP in ASPP2-depleted cells. Moreover, the mRNA expression of YAP was also found to be decreased in ASPP2-deficient cells. This result is further supported by the positive correlation of ASPP2 and YAP at mRNA levels in pancreatic cancer patient samples. Given that transcription regulation-associated GO terms (GO:0000122, GO:0045944, GO:0006366, etc.) were identified among the most significantly changed biological processes, and how YAP regulation at the mRNA level is still poorly understood, it would be exciting to unravel the mystery of ASPP2-mediated control of YAP mRNA. We plan to delve into more details on this through a well-performed ChIP-seq analysis in the future.

## 5. Conclusions

This study reveals the phosphorylation mechanism of ASPP2 during mitosis and demonstrates that ASPP2 depletion does not promote (yet it does repress) the proliferation of pancreatic cancer cells. An unbiased RNA-seq analysis was performed and pinpointed YAP as a central player downstream of ASPP2 that affects proliferation-associated gene transcription. Taking into consideration the current as well as previous findings, ASPP2 functions should not be stringently defined based on simple tumor-suppressing and tumor-promoting dichotomy, but rather, ASPP2 should be considered a sophisticated protein and may have multi-faceted roles in cancer.

Overall, this study focused on investigating how ASPP2 phosphorylation is regulated during mitosis and the function of ASPP2 in pancreatic cancer growth. Our findings demonstrate that ASPP2 is phosphorylated by CDK1 at S562 and S704 during mitosis and is required for pancreatic cancer cell proliferation in vitro and in vivo. We also analyzed the transcriptional landscape altered by ASPP2 and centered YAP as the key player in it. Collectively, results strongly indicate that ASPP2 is a potential target for abating tumor cell growth in pancreatic cancer.

## Data Availability

All data generated in this study are available upon request.

## Supplementary Materials

The following supporting information can be downloaded at: https://www.mdpi.com/article/10.3390/cancers15225424/s1.
