# Supplementary material for: ASPP2 Is Phosphorylated by CDK1 during Mitosis and Required for Pancreatic Cancer Cell Proliferation
Source: Cancers (Basel). 2023 Nov 15;15(22):5424. doi: 10.3390/cancers15225424 (PMC10670399; doi:10.3390/cancers15225424)

Figure 1A

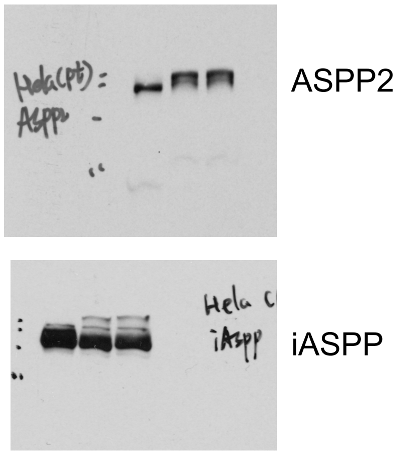

Figure 1B

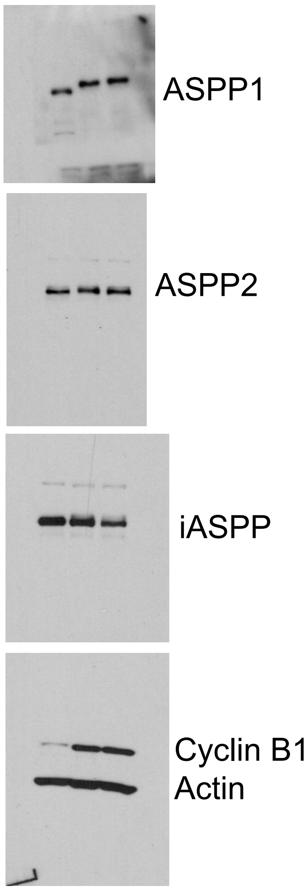

Figure 1C

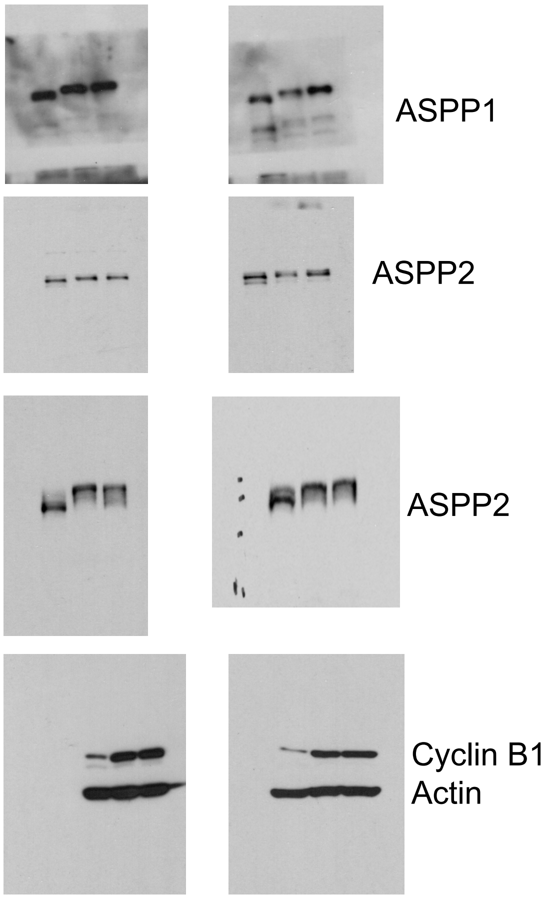

Figure 1D

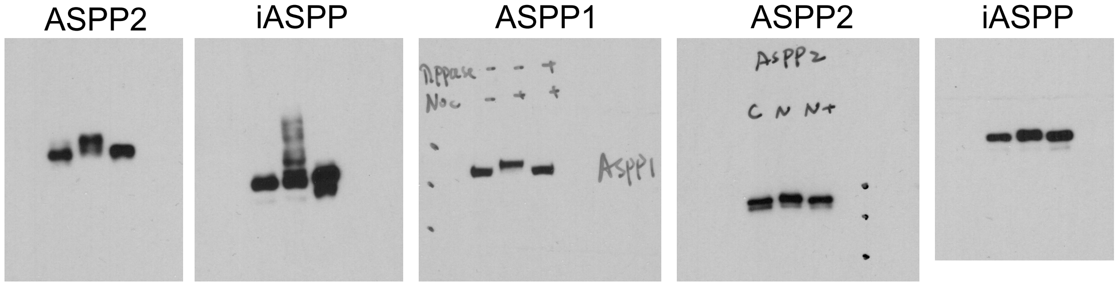

Figure 1E

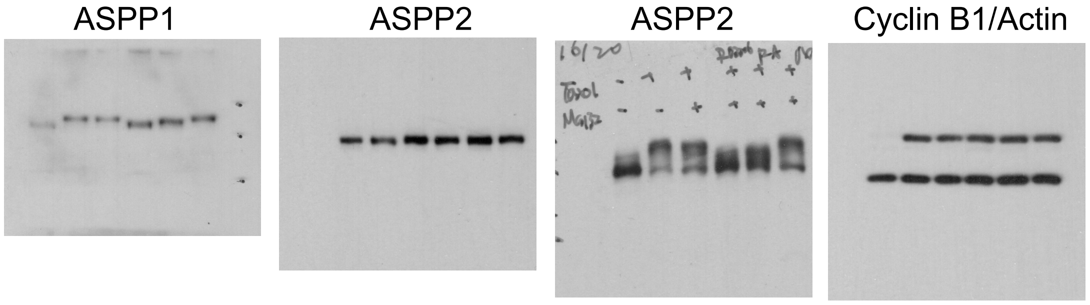

Figure 2B

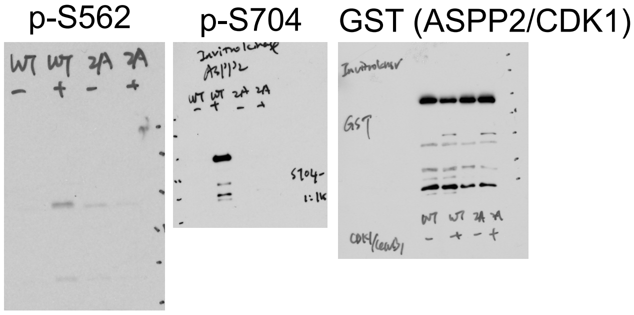

Figure 2C

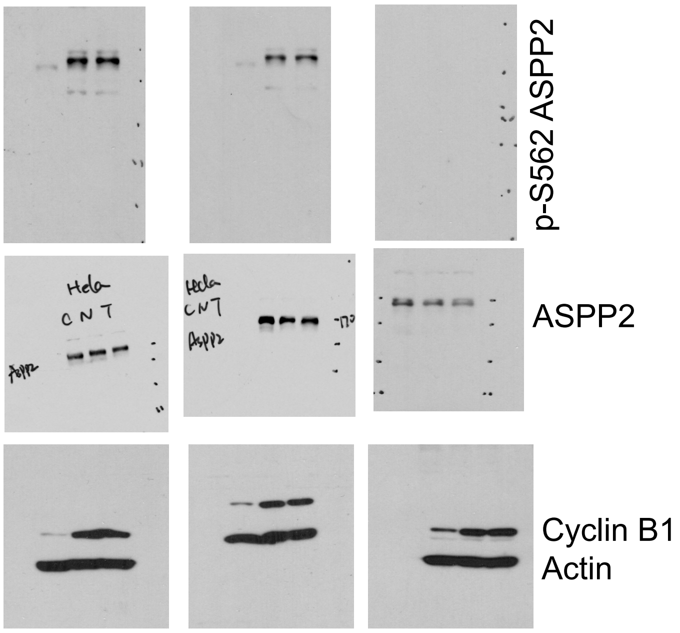

Figure 2D

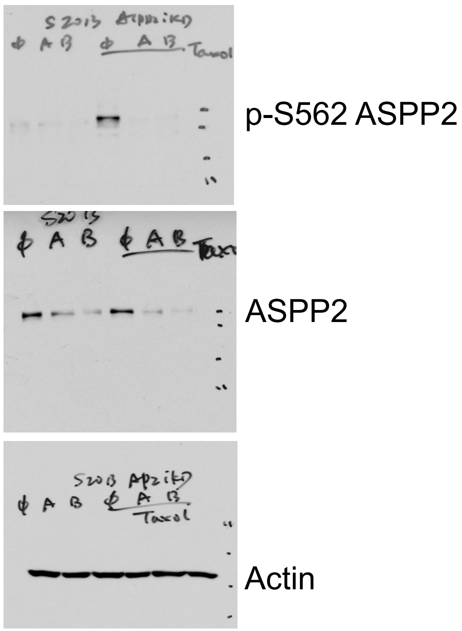

Figure 2E

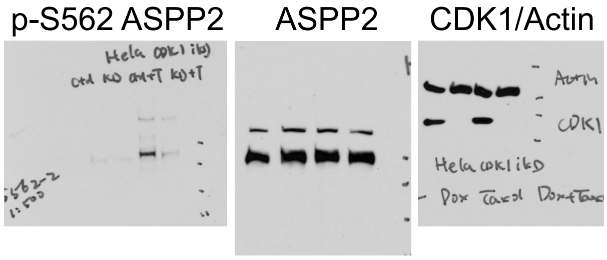

Figure 2F

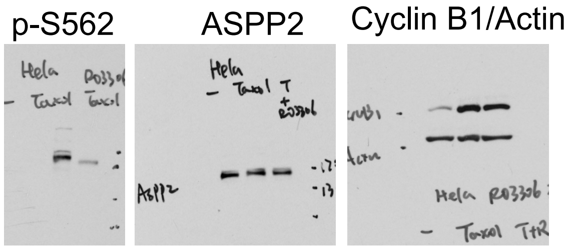

Figure 3C

ASPP2

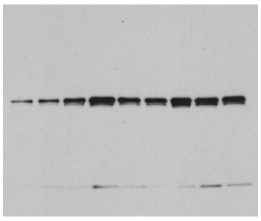

Actin

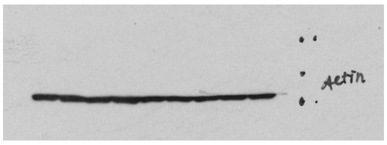

Figure 4A

ASPP2

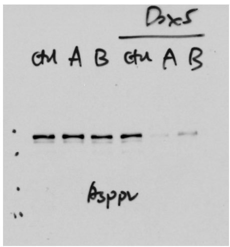

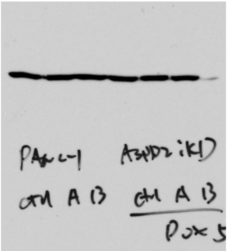

Actin

Figure 4B

ASPP2

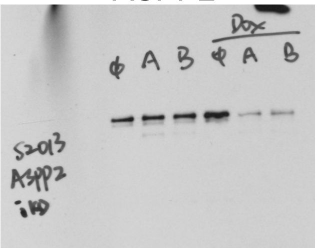

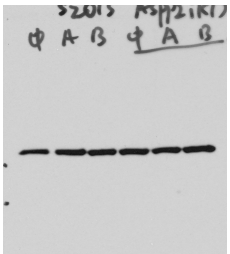

Actin

Figure 4H

ASPP2

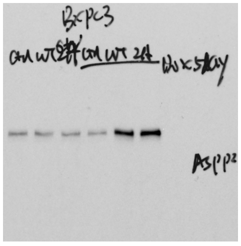

Actin

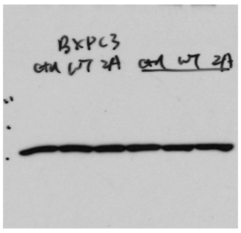

Figure 4I

ASPP2

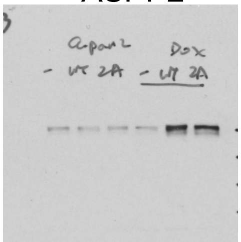

Actin

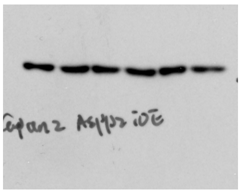

Figure 6D

ASPP2

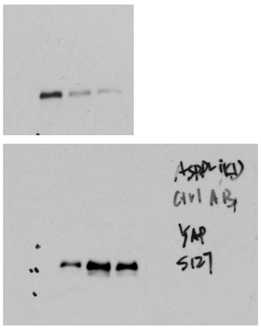

YAP

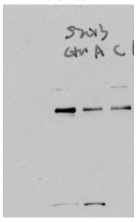

Actin

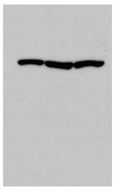

Supplement: Supplementary file 1 [file cancers-15-05424-s001.zip › cancers-2664200-original-images.pdf]
